# The Use of Technology to Protect the Health of Athletes During Sporting Competitions in the Heat

**DOI:** 10.3389/fspor.2019.00038

**Published:** 2019-10-03

**Authors:** Borja Muniz-Pardos, Shaun Sutehall, Konstantinos Angeloudis, Jonathan Shurlock, Yannis P. Pitsiladis

**Affiliations:** ^1^Growth, Exercise, Nutrition and Development (GENUD) Research Group, University of Zaragoza, Zaragoza, Spain; ^2^Division of Exercise Science and Sports Medicine, University of Cape Town, Cape Town, South Africa; ^3^Collaborating Centre of Sports Medicine, University of Brighton, Eastbourne, United Kingdom; ^4^Sheffield Teaching Hospitals, Sheffield, United Kingdom; ^5^Sciences, University of Rome “Foro Italico”, Rome, Italy; ^6^International Federation of Sports Medicine (FIMS), Lausanne, Switzerland

**Keywords:** hot environment, heat stroke, exertional heat illness, olympics, world championships, health, athlete protection

## Abstract

During the 2019 IAAF World Championships in Athletics in Doha and the 2020 Olympic Games in Tokyo, minimum daily temperatures are expected to be in excess of 30°C. Due to the metabolic demands of the sporting events and the high environmental temperatures, the risk of exertional heat stroke (EHS) is high. Careful planning by event organizers are needed to ensure that athletes are protected from irreversible long-term health damage, or even death during sporting competitions in the heat. Efforts typically have included standard medical plans, equipment, protocols, and expert medical teams. In addition, the importance of responding quickly to a hyperthermic athlete cannot be understated, as minimizing treatment time will greatly improve the chances of full recovery. Treatment time can be minimized by notifying medical personnel about the health status of the athlete and the extent of any pre-competition heat acclimatization. Technology that allows the live transmission of physiological, biomechanical, and performance data to alert medical personnel of potential indicators of EHS should be considered. Real time monitoring ecosystems need to be developed that integrate information from numerous sensors such as core temperature-monitoring “pills” to relay information on how an athlete is coping with competing in intense heat. Medical/support staff would be alerted if an athlete's responses were indicating signs of heat stress or EHS signs and the athlete could be withdrawn under exceptional circumstances. This technology can also help provide more rapid, accurate and dignified temperature assessment at the road/track side in medical emergencies.

## Introduction

The 17th IAAF Track and Field World Championships will be held in Doha (Qatar) from 27th September to 6th October, 2019. This will be one of the most anticipated sporting events of the 2019 calendar, with an estimated 3,500 athletes, 40,000 international guests, and more than 2,000 media personnel. In addition to being one of the most hotly anticipated events of the year, daily and peak temperatures are expected to be ~32 and 40°C, respectively, with a relative humidity of ~60% (Time and Date, 2018).

While the relevant governing bodies and authorities are expected to take all essential measures to avoid athletes competing in extreme heat (e.g., midnight marathon and the removal of all morning sessions), the reality remains that the minimum daily temperature during events such as the marathon is likely to be ~30°C [as it was in 2018 during the same day (Time and Date, 2018)]. The impact of exercising under such extreme environmental conditions will increase the likelihood of developing exertional heat illness (EHI), which is associated with a core body temperature above 40°C (Knochel, 1974). The symptoms of EHI include coordination difficulties (Hubbard and Armstrong, 1988), reduced cognitive function (Lieberman et al., 2005), impairment in endurance performance (Galloway and Maughan, 1997; Nybo et al., 2014), and eventually exertional heat stroke (EHS) followed by collapse (Kenefick and Sawka, 2007). There are cases of fatal EHS in environmental conditions similar to those which will be experienced in Doha and Tokyo. For example, several cases of fatal EHS have been reported in novice runners performing long-distance road races (>10 km) at 24–26°C and a relative humidity ranging from 60 to 66% (Hanson and Zimmerman, 1979), and also in a runner performing sprint sets at 29°C (relative humidity not reported) (Graber et al., 1971). There are indications that the greatest risk for EHS occurs when the wet bulb globe temperature (WBGT) surpasses 28°C during high-intensity exercise and/or strenuous exercise that lasts more than an hour (Armstrong et al., 2007). The WBGT is a climatic index used worldwide by event organizers, and it is calculated from the air temperature, humidity, mean radiant temperature, and wind speed (Casa, 2018). This index applies a weighted average between the natural wet-bulb temperature (which indicates the true capacity of the air to evaporate water according to its relative humidity and velocity), the dry bulb temperature, and the solar radiation (globe temperature; Casa, 2018). As an environmental indicator, this index does not account for metabolic heat production or clothing, and therefore cannot predict heat dissipation (Sawka et al., 2011). Therefore, EHS can occur even during apparently normal environmental conditions, following WBGT indications (Casa et al., 2015), which questions the suitability of using an environmental index only in sporting events. Hosokawa et al. (2018) have recently developed 36-year (1980–2016) modeled climatologic datasets for the Japanese and Qatar venues for the upcoming events, showing the hourly WBGT.

Time is also critical in EHS management as an athlete is more likely to suffer long-term disability if treatment does not commence within 30 min and the risk of death increases if treatment is delayed by more than 60 min (Adams et al., 2015). A recent case that highlights the importance of a rapid intervention during EHS is the collapse of the Scottish marathoner Callum Hawkins during the 2018 Commonwealth Games in Queensland (Australia) in an environmental temperature of 30°C. Callum was leading the race and was 2 km from the finish line when he first collapsed. Although medical assistance arrived several minutes after his collapse, the event organizers were heavily criticized for insufficient medical care (The Guardian, 2018). This unfortunate event warrants the introduction of additional measures to those normally in place in competitions in warm-hot weather conditions, especially considering EHS is the second largest cause of death in sport, after cardiovascular events (Casa and Stearns, 2016). Demartini et al. (2015) examined 274 cases of EHS with body temperatures above 40°C and showed that immediate temperature evaluation and CWI treatment resulted in 100% survival. This illustrates the importance of a prompt recognition and immediate treatment during EHS. Moreover, two major international events are planned within the next year (i.e., the 2019 IAAF World Championships in Doha and the 2020 Olympic Games in Tokyo) which highlights the need to characterize the thermoregulatory responses of athletes competing in high-risk sports (e.g., race walking, triathlon, marathon running) to effectively protect the health of athletes and adapt current policies. A recent report documented 57 deaths and more than 18,000 people taken to hospitals due to heat-related medical issues over the week starting the 29th July, 2019 in Japan; the exact date of the Tokyo Olympics next year (The Japan Times, 2019). This is a timely reminder of the magnitude of the potential risks if organizers, athletes, spectators and the general population are not fully informed of the importance of taking appropriate preventive measures. Additionally, many of the athletes competing in Doha 2019 will attend the 2020 Olympic Games in Tokyo next summer. These athletes should also be aware that an EHS event requires full recovery prior to returning to training. Concerted efforts to enhance medical provision will also provide an essential legacy for future top-level competitions such as the Olympic Games planned in hot and humid climes of Paris (2024) and Los Angeles (2028).

Although the safest “return to play” strategy after an EHS event remains to be determined, previous research suggests resting up to 21 days before returning to exercise (Casa et al., 2015). However, there are reports stating that up to 60 days of rest may be necessary (Lopez et al., 2018). In the case of incomplete recovery from EHS, the athlete may become heat intolerant, exhibiting an abnormally high physiological response to exercise in the heat (Casa, 2018). Potential residual effects of EHS occurring in Doha, will likely compromise the athlete's preparation and performance in the 2020 Tokyo Olympics. The long-term adverse effects in individuals who have suffered from EHS depend directly upon the duration of hyperthermia, the intensity of shock, the rate of cooling, and the prompt recognition and treatment of associated severe complications (Shapiro and Seidman, 1990). It is worth noting that body temperature at collapse is usually between 41.1 and 43.3°C, and athletes are likely to have been exercising above critical body temperature without apparent signs of EHI for an unknown amount of time when loss of consciousness is observed (Adams et al., 2015). Those patients who survive severe EHS often show cerebellar damage and cerebellar ataxia with marked dysarthria and dysmetria (Yaqub, 1987; Royburt et al., 1993). In fact, residual neurological damage has been observed in up to 20% of EHS survivors (Bouchama and Knochel, 2002). Accordingly, previous research presented persistent neurological deficits and personality changes 3 years (Lin et al., 1991) and 11 years (Mehta and Baker, 1970) after EHS.

**Figure 1:**
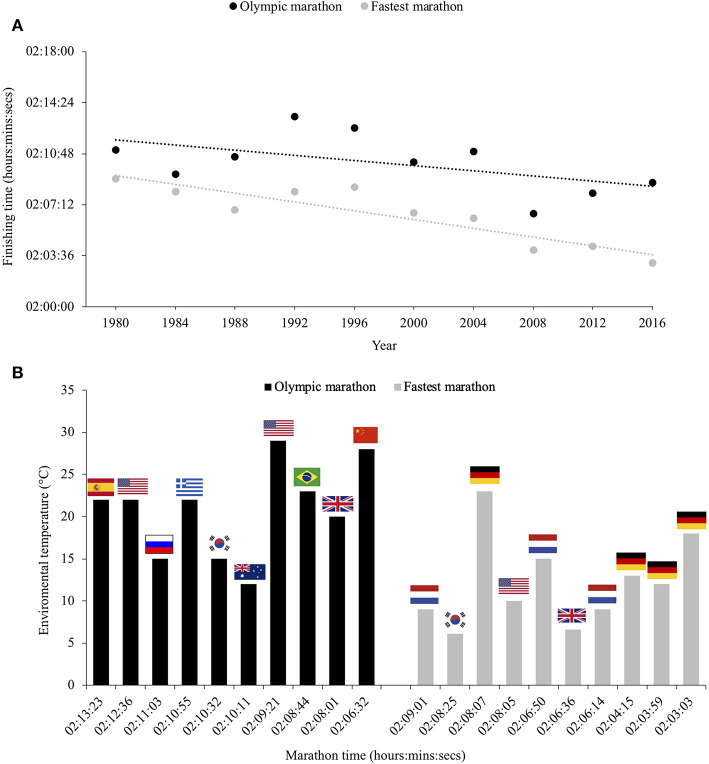
Comparison of the fastest men's marathon times during Olympic year with the winning times at the Olympic marathons over the past four decades **(A)** and the temperature disparity between these marathons **(B)**.

The Summer Olympics over the past three decades have been held in July or August, recognized as an ideal time for television networks to cover the event, but highly problematic for organizers who must protect athletes from the hot weather in several of the host cities such as Los Angeles (1984), Athens (2004), Beijing (2008), and Rio de Janeiro (2016). The hot and humid conditions can negatively impact sporting performance, and this effect is most pronounced during endurance events as can be illustrated by comparing the fastest men's marathon times during an Olympic year with the winning time at the Olympic marathon (Figure 1). While there are other contributing factors to the slower Olympic marathon times (e.g., slower courses, tactical races, absence of pace makers, medals on offer rather than prize money), the hot and humid conditions clearly play a major role.

There are numerous strategies that can be adopted by the athlete prior to and during competition to attenuate the rise in core temperature, although heat acclimation/acclimatization appears to be the most beneficial. Acclimatization differs from acclimation in that the adaptive characteristics are augmented in a natural climate or environment, whereas the stimuli for acclimation is artificially induced, typically through an environmental chamber where ambient conditions are altered (Racinais et al., 2015). The primary adaptations following heat acclimatization include a decrease in core temperature (Périard et al., 2015), increased control of cardiovascular function (Armstrong and Maresh, 1991), and increased sweat rate with earlier onset and more dilute sweat (Mack and Nadel, 1996), all of which facilitate an improvement in performance in both hot and temperate environments (Lorenzo et al., 2010). There are multiple factors to consider in order to optimize heat acclimatization (Armstrong and Maresh, 1991), and protocols range from short (e.g., <7 daily exposures) to long (e.g., >15 daily exposures) durations. However, the maximal adaptation to a specific environment will result from simulating the environmental and work characteristics of the real environment one wishes to perform (Périard et al., 2015). Therefore, it is advisable that all athletes competing in endurance events (especially in events longer than 1,500 m) in extremely hot conditions such as those expected during Doha 2019 and Tokyo 2020, should undertake a long-term (>15 daily exposures) heat acclimatization, while exercising at intensities similar to that expected during the competition (Périard et al., 2015). Casa et al. (2009) highlighted the need for exercise duration and intensity to be gradually increased to avoid potential EHI during heat acclimatization. For optimal heat acclimatization to occur, a core temperature above 38.5°C is necessary (Patterson et al., 2004; Garrett et al., 2009). However, until now, such controlled hyperthermia during exercise required a laboratory setting to monitor core temperature in real time, albeit acclimatization is possible by prescribing the training intensity in hot ambient conditions using heart rate (Périard et al., 2015). In addition to heat acclimatization, there exists other, acute methods of attenuating rises in core temperature, known as “cooling strategies” before or during the sporting event (Ross et al., 2013). However, athletes taking these measures are still at risk of suffering from EHS, especially considering the difficulty of identifying signs of EHS.

Given the dangers associated with competing in extreme hot conditions and the likelihood that this occurrence will become more common, there is an urgent need to develop and implement the latest technologies to further our understanding of the thermoregulatory and physiological responses of elite athletes competing in extreme environmental conditions. The focus of this perspective is to examine potentially useful future innovations to prevent EHS. One such technological development allows the live transmission of physiological, biomechanical, and performance data that is able to alert medical personnel to abnormal perturbations of athletes and officials and save precious minutes in a medical emergency. Therefore, the aim of this perspective in conjunction with other related papers in this edition, is to raise awareness within the current policies to safeguard the health of athletes through innovative technologies.

## Technological and Practical Approaches

The application of wearable technology in recreational and elite sport is replacing traditional laboratory testing since wearables allows the assessment of numerous physiological and biomechanical responses and performance in real-life situations. However, a recent review highlighted the lack of quality assessment procedures (i.e., rigorous validity and reliability tests) that demonstrate the efficacy of an ever-increasing number of wearables (Peake et al., 2018). This review highlighted that only 5% of the commercially available wearables have been suitably validated, which make it difficult for athlete, technical or medical staff to identify high-quality and useful wearables (Peake et al., 2018). Notably, the most severe form of EHI (i.e., EHS) cannot be studied in the laboratory due to the risk of permanent damage to participants. As a consequence, the use of wearable sensors to measure body temperature has increased to enable data collection from individuals competing in hot ambient conditions. Monitoring of skin temperature has been divided into two subcategories (Tamura et al., 2018): the tach-type thermometers which are sensitive non-disposable wearable prototype devices that are applied directly to the human body using a variety of materials such as Tempdrop (Tempdrop, 2019), Ran's night (Ran's Night, 2019), and iFiever (Vipose Smart Thermometer, 2019). The second category is the patch-type wearables, which are wireless and electronic miniaturized disposable sensors that are attached to the skin surface by the application of an adhesive patch to any part of the body such as VitalConnect (VitalConnect, 2019), FeverFrida (FeverFrida the iThermonitor, 2019), STEMP (Smart Temperature Patch, 2019), TempTraq™ (TempTraq Landing, 2019), Fever Scout (Fever Scout, 2019), and Fever Smart (Feversmart). These allow the continuous monitoring of skin temperature, human skin hydration through the assessment of thermal conductivity, as well as blood variables (Tamura et al., 2018). These sensors are usually made from plastic materials or a small amount of silicon, which is used as a membrane around the core of the sensors. All these sensors are designed to measure skin temperature (Tamura et al., 2018), but none adequately reflect core temperature. There is also an important gap in the scientific literature examining the validity and reliability of such devices, which also questions their use (Casa, 2018). It is essential, therefore, that independent research institutions are set up to regulate the quality standards of wearable technologies to support entry into the market when sufficient validity and reliability has been demonstrated (Düking et al., 2018). Given these and other limitations, but also the need to accurately measure core temperature *in situ* in the competing athlete, a number of technological developments are needed. One such direct method to continuously monitor core temperature is the use of an ingestible thermometer pill. This method is gaining popularity in sport and is already extensively used in numerous ambulatory settings (Casa, 2018). There are at least three different ingestible pills systems in production and development: HQInc, Philips/Respironics, and BodyCap (Casa, 2018). Of these, the ingestible pill manufactured by BodyCap is the most miniaturized core temperature-monitoring sensor (1.7 g), which facilitates its tolerability and use. The pill is wirelessly connected to an external monitor device, although the main limitation of this technology is that the pill needs to be in close proximity to the monitor to enable live feedback and transmission. To date, none of these commercially available pill systems can provide the desired real time *in situ* assessment.

**Figure 2:**
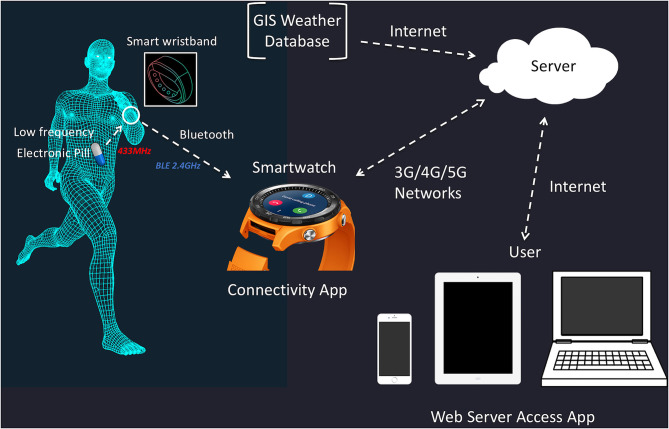
Smart activity and temperature monitor to enhance safety during sporting events with particular reference to athletes, officials, and workforce at increased risk. Adapted from Düking et al. (2018) with permissions from Wolters Kluwer Health, Inc.; License number: 4654951032268.

Given the need to better protect the athlete exercising in extreme conditions (or other personnel such as military and members of the emergency services), we are attempting to develop live-transmitting technology that allows the tracking of multidisciplinary data within a single application. This innovation is a system that provides live feedback of land and air temperature, heart rate, and a range of physiological and biomechanical parameters facilitated through a Cloud-based portal allowing the athlete support/medical team to view the data on a desktop, tablet, or a smartphone in real time anywhere with internet access or mobile access (Düking et al., 2018; Muniz-Pardos et al., 2018; Figure 2). An early prototype version of this application was recently trialed in elite marathoners training at altitude in Iten (Kenya) with the addition of sensors that also identified foot strike patterns (e.g., pitch angles, strike angles, contact times), transmitted second by second to a portal database (Muniz-Pardos et al., 2018). This feasibility study illustrated the capacity of wearable technology to monitor fatigue, injury or even EHS signs through biomechanical assessment. Given the threat of the internet, WIFI, and other traditional networks being compromised or hacked during high profile sporting events such as the Olympics, alternative approaches to transmit data and communicate are needed. Notably, Firechat and Bluetooth Bridgefy Apps have recently been used with much success by protesters to avoid being traced by authorities (BBC News, 2019). The mobile network, especially as 5G becomes more widely available, represents, for now at least, the preferred option to transmit data and communicate effectively.

The development of an ecosystem that allows the real time assessment of body temperature but also other physiological (e.g., cardiorespiratory responses) and biomechanical responses (e.g., imbalances and irregular strides of the lower limbs) of an athlete, could undoubtedly help protect athletes from EHS by helping alert medical staff of impending conditions such hyperthermia. For example, wireless foot-worn inertial sensors (FWIS) with a wireless foot insole pressure system (FIPS) and dedicated signal processing algorithms have recently been developed that can detect spatiotemporal variables (e.g., contact time, stride frequencies, ground reaction forces, and strike angles) of the foot and running mechanics (Mariani et al., 2013; Falbriard et al., 2018; Muniz-Pardos et al., 2018; Peake et al., 2018). This quantification of the kinetic and kinematic modulation of the lower limbs (i.e., loading rates, impact forces) is able to provide unique insights into the foot biomechanical characteristics and therefore running mechanics of athletes across different running and environmental conditions. It is known that alterations in running technique can substantially influence the neuromuscular and kinematic characteristics of running (Lieberman et al., 2010; Udofa et al., 2019). There is also some evidence that foot biomechanical characteristics such as contact and swing time can be modulated during strenuous exercise in response to heat stress (Girard et al., 2016). These technological advances will undoubtedly aid scientists, physicians, and athletes to make more informed decisions about the effectiveness of therapeutic methods, preventive interventions, and other medical approaches.

Another convenient wearable recently launched is the utilization of smart bottles linked to a smart device to track fluid intake. The smart drinking device launched by a major drinks company, incorporates an auto spout, auto seal, and achieves a high flow rate and is able to communicate digitally with a “band aid-like” sweat patch claimed to track the hydration status of the athlete (Burke, 2019). The utilization of such technology along with other wearable technology transmitting numerous types of data in real time, will inevitably become the norm at major sporting events as international sporting federations seek to make their sport more interesting and accessible to their audiences. These technological developments can also be harnessed to help protect the health of athletes (and officials) from numerous conditions not confined only to EHS but can include many other health-related conditions such as concussion and cardiac sudden death and help make more objective and informed decisions about leaving and/or returning to the field of play. The implementation of real time technology would potentially permit the earlier identification and more effective treatment management of athletes by medical personnel during a medical emergency.

It is unquestionable that within a decade or even less, wearables will be worn at all times and data collected will be fed into machine-learning algorithms to monitor vital signs, identify abnormalities and track treatments, so that medical problems can be detected earlier (Xu et al., 2019). An example of this technology is the novel self-applied wearable electrocardiogramme patch, which can monitor heart dynamics for 14 days. Despite only limited data, there are some encouraging results that show this wearable to be more effective in detecting signs of atrial fibrillation than occasional medical monitoring (Steinhubl et al., 2018). Tandon and de Ferranti (2019) have recently described the capacity of wearable sensors to alert the medical team of cardiovascular disease from real time continuous physiological data monitoring in an infant population. Such technological developments will transform the health-care system from hospital-based interactions to more continuous home-based care (Tandon and de Ferranti, 2019). Wearable chemical sensors to monitor body fluids are increasingly being described (Matzeu et al., 2015; Ray et al., 2019). In particular, blood monitoring has been extensively used in the medical field for the last few years to measure a number of parameters, although recent research recommend the use of non-invasive chemical analysis of biofluids such as sweat, tears, saliva, or interstitial fluids, providing minimal risk of harm or infection and are generally more user friendly (Kim et al., 2019). Of these easily accessible body fluids, sweat has been particularly amenable to the detection of sodium (Bandodkar et al., 2014), glucose (Lee et al., 2017), and lactate levels (Anastasova et al., 2017). All these parameters are potentially interesting to the sports physician/exercise scientist as markers of electrolyte balance and energy homeostasis. These necessary advances are encouraging leading many experts in the field to contend that we are facing the first generation of “biointegrated sensors,” which will require close collaborations between materials and device engineers, scientists, and medical professionals to optimize the utility of this technology (Ray et al., 2019; Xu et al., 2019). The power of this technology can also be integrated into the fight against doping by the introduction of remote monitoring of molecules in sweat or blood. Such advances would also generate exciting new possibilities for checking an athlete's metabolism and aid the policing of doping in sport (The Guardian, 2016).

## Concluding Remarks

Given the two upcoming high-profile sporting events conducted in extremely hot ambient conditions (Doha 2019 and Tokyo 2020), it is essential that event organizers and those responsible for the health of athletes, officials, work staff, and other populations at risk of EHS, are well-informed and prepared. Due to the extreme environmental conditions, it is critical for medical staff to recognize early the symptoms of EHS and to quickly and effectively intervene. The monitoring of an athlete's physiological responses such as core temperature, hydration status, and relevant biomechanical parameters and its transmission in real time to support/medical personnel will accelerate the recognition and treatment of any athlete suffering from EHS. The medical teams would be better equipped to recognize earlier EHS if they could be informed of the acclimatization strategies, history of EHI, current viral illnesses, and sleep diaries of athletes. These measures and technological approaches seem necessary to protect athletes and other populations at risk to perform at their best while minimizing the risk of serious illness or even death at these and future sporting events conducted in extreme environmental conditions.

## Data Availability Statement

All datasets generated for this study are included in the manuscript.

## Author Contributions

All authors contributed significantly to this manuscript.

### Conflict of Interest

YP is the founding member of the Sub2 Foundation (www.sub2hrs.com) that is affiliated to a company that is a minor shareholder of Maurten AB. The remaining authors declare that the research was conducted in the absence of any commercial or financial relationships that could be construed as a potential conflict of interest.
